# Anti-allodynic effect of Buja in a rat model of oxaliplatin-induced peripheral neuropathy via spinal astrocytes and pro-inflammatory cytokines suppression

**DOI:** 10.1186/s12906-017-1556-z

**Published:** 2017-01-14

**Authors:** Yongjae Jung, Ji Hwan Lee, Woojin Kim, Sang Hyub Yoon, Sun Kwang Kim

**Affiliations:** 1 Department of Clinical Korean Medicine, Graduate School, Kyung Hee University, Seoul, 02447 Republic of Korea; 2 Department of Science in Korean Medicine, Graduate School, Kyung Hee University, Seoul, 02447 Republic of Korea; 3 Department of Physiology, College of Korean Medicine, Kyung Hee University, Seoul, 02447 Republic of Korea; 4 Department of Digestive system of Internal Medicine, College of Korean Medicine, Kyung Hee University, Seoul, 02447 Republic of Korea

**Keywords:** Astrocytes, Buja, Cold allodynia, Oxaliplatin, Pro-inflammatory cytokines, Spinal cord

## Abstract

**Background:**

Oxaliplatin, a widely used anticancer drug against metastatic colorectal cancer, can induce acute peripheral neuropathy, which is characterized by cold and mechanical allodynia. Activation of glial cells (e.g. astrocytes and microglia) and increase of pro-inflammatory cytokines (e.g. IL-1β and TNF-α) in the spinal cord play a crucial role in the pathogenesis of neuropathic pain. Our previous study demonstrated that Gyejigachulbu-Tang (GBT), a herbal complex formula, alleviates oxaliplatin-induced neuropathic pain in rats by suppressing spinal glial activation. However, it remains to be elucidated whether and how Buja (Aconiti Tuber), a major ingredient of GBT, is involved in the efficacy of GBT.

**Methods:**

Cold and mechanical allodynia induced by an oxaliplatin injection (6 mg/kg, i.p.) in Sprauge-Dawley rats were evaluated by a tail immersion test in cold water (4 °C) and a von Frey hair test, respectively. Buja (300 mg/kg) was orally administrated for five consecutive days after the oxaliplatin injection. Glial activation in the spinal cord was quantified by immunohistochemical staining using GFAP (for astrocytes) and Iba-1 (for microglia) antibodies. The amount of spinal pro-inflammatory cytokines, IL-1β and TNF-α, were measured by ELISA.

**Results:**

Significant behavioral signs of cold and mechanical allodynia were observed 3 days after an oxaliplatin injection. Oral administration of Buja significantly alleviated oxaliplatin-induced cold and mechanical allodynia by increasing the tail withdrawal latency to cold stimuli and mechanical threshold. Immunohistochemical analysis showed the activation of astrocytes and microglia and the increase of the IL-1β and TNF-α levels in the spinal cord after an oxaliplatin injection. Administration of Buja suppressed the activation of spinal astrocytes without affecting microglial activation and down-regulated both IL-1β and TNF-α levels in the spinal cord.

**Conclusions:**

Our results indicate that Buja has a potent anti-allodynic effect in a rat model of oxaliplatin-induced neuropathic pain, which is associated with the inhibition of activation of astrocytes and release of pro-inflammatory cytokines in the spinal cord. Thus, our findings suggest that administration of Buja could be an alternative therapeutic option for the management of peripheral neuropathy, a common side-effect of oxaliplatin.

**Electronic supplementary material:**

The online version of this article (doi:10.1186/s12906-017-1556-z) contains supplementary material, which is available to authorized users.

## Background

Oxaliplatin is a third-generation platinum-based chemotherapy drug, which is widely used as the first-line treatment of metastatic colorectal cancer [1–5]. Despite its efficacy against the tumor, it has serious neurotoxicity, a dose-limiting side effect. This neurotoxicity is characterized by paresthesia and dysesthesia in the hands and feet [6], and about 85 to 95% of patients rapidly develop significant acute neuropathic pain without motor dysfunction shortly after an oxaliplatin infusion [7, 8]. Several drugs (e.g. gabapentin and duloxetine) are recommended to mitigate this side effect [9–11]. Unfortunately, these analgesics cause another side effects, such as somnolence and nausea [12].

Activation of glial cells, such as astrocytes and microglia, has been observed in the lumbar spinal cord in animal models of peripheral neuropathic pain [13–17]. Upon activation, astrocytes and microglia release a variety of substances that enhance the transmission of pain, such as pro-inflammatory cytokines [18, 19]. Both interleukin (IL)-1β and tumor necrosis factor-α (TNF-α) enhance the spontaneous excitatory post-synaptic currents frequency of spinal dorsal horn neurons [20]. In animal models of chemotherapy-induced peripheral neuropathy (CIPN), the causal relationship between glial activation and neuropathic pain has also been reported [21–24]. Oxaliplatin treatment lowered the pain threshold combined with a significant increase in the number of GFAP (astrocyte) and Iba-1 (microglia) immunoreactive cells in the spinal dorsal horn [21, 22]. In addition, a single injection of oxaliplatin induces spinal glial activation coincident with pain behaviors like cold and mechanical allodynia [25].

Buja, a processed Aconiti tuber, is one of the frequently used herbal medicine in several diseases [25–27]. Previous articles have reported its analgesic effect on different kinds of neuropathic pain, such as diabetic neuropathy and postherpetic neuralgia [28, 29]. Buja inhibited neuropathic mechanical allodynia by suppressing the activation of spinal astrocytes in a nerve injury model [26]. Also, a recent clinical study has reported that Buja reduced neuropathic pain in oxaliplatin-treated colorectal cancer patients [30]. Gyejigachulbu-tang (GBT), which is composed of Cinnamomi Cortex (Yukgye; in Korean), Peoniae Radix (Jakyak), Atractylodis Lanceae Rhizoma (Bokryeng), Ziziphi Fructus (Saenggang), Glycyrrhizae Radix (Gamcho), Zingiberis Rhizoma (Gungang) and Aconiti Tuber (Buja), showed a potent analgesic effect against oxaliplatin-induced peripheral neuropathy in rats. Such effect of GBT is associated with deactivation of spinal astrocytes and microglia [25].

In the present study, we investigated whether Buja relieves oxaliplatin-induced cold and mechanical allodynia in rats, and if so, whether such anti-allodynic effect of Buja is related to the modulation of glial activation and pro-inflammatory cytokines in the spinal cord.

## Methods

### Animals

Young adult male Sprague-Dawley rats (Daehan Biolink, Chungbuk, Korea), weighing approximately 200–220 g, at the beginning of the experimental procedure were used. Animals were housed in cages (3–4 rats per cage), and fed with water and food ad libitum. The room was maintained with a 12 h-light/dark cycle (a light cycle; 08:00–20:00, a dark cycle; 20:00–08:00) and kept at 23 ± 2 °C. All animals were acclimated in their cages for 1 week prior to any experiments. All procedures involving animals were approved by the Institutional Animal Care and Use Committee of Kyung Hee University (KHUASP(SE)-15-088) and were conducted in accordance with the guidelines of the International Association for the Study of Pain [31].

### Drug administration

Oxaliplatin (Sigma-Aldrich, St Louis, MO, USA) was delivered at an amount of 6 mg/kg [32, 33], dissolved in 5% glucose (Sigma-Aldrich) solution at a concentration of 2 mg/ml. Oxaliplatin was administered intraperitoneally (i.p.). Control animals received an equivalent volume of 5% glucose solution i.p. as a vehicle.

Buja (Bushi in Japanese; TJ-3023, Tsumura Co. Ltd., Ibaraki, Japan) was obtained by a generous gift from Prof. Schuichi Koizumi (Department of Neuropharmacology, Faculty of medicine, University of Yamanashi). The quality of Buja is strictly controlled by the manufacturer. Buja was suspended and diluted with distilled water (30 mg/ml, 300 mg/kg, approximately 2–2.2 ml/rat) [26]. Our preliminary study using several doses of Buja confirmed that 300 mg/kg was the optimal concentration. Buja was treated orally for 5 consecutive days after an oxaliplatin injection. Equivalent volume of distilled water (DW) was administered to control animals.

Animals were arbitrarily divided into 4 groups: Vehicle + DW, group1; Vehicle + Buja, group2; Oxaliplatin + DW, group3; Oxaliplatin + Buja; group4.

### Behavioral tests

For assessment of oxaliplatin-induced neuropathic pain before and after Buja administraion, cold and mechanical test were performed. Cold allodynia were determined by cold immersion test as previously described [34, 35]. In brief, rats were placed into an acrylic cylinder holder with the tail protruding and were adapted to the testing environment at least 30 min prior to testing. After immersing the tail in 4 °C water, the tail withdrawal latency (TWL) was measured with a 15 s cut-off time. This test was repeated 5 times at 5 min intervals to prevent tissue damage. The average of each latency was used to represent cold allodynia (i.e. the shorter latency was considered the more severe cold allodynia). Mechanical allodynia was evaluated the withdrawal response of tail using a series of von Frey filaments (bending forces to 0.4, 0.6, 1.0, 2.0, 4.0, 6.0, 8.0 and 15.0 g; equivalent in log units: 3.61, 3.84, 4.08, 4.31, 4.56, 4.74, 4.93 and 5.18; Stoelting, IL, USA), as previously described [25]. Using the up-down method, the 50% withdrawal threshold was determined [36]. Rats were immobilized in an acrylic cylinder holder as mentioned above. Test was initiated with a filament having 2.0 g bending force. When withdrawal of the tail was observed during or right after stimulation, this was considered as a positive response. The filament with the next lower bending force was applied after a positive response was observed, whereas the filament with the next higher bending force was applied when there was no response (i.e. negative response). This procedure continued until the sixth von Frey filament stimulation from the first stimulation (2.0 g) or until the third change of response (positive or negative) was observed. The extreme values of series of von Frey filaments were set as a cut-off value. These responses were converted into 50% threshold value using the following formula: 50% threshold = Xf + κδ (Xf is the value of the final von Frey filament [log units], κ is the correction factor from calibration table, and δ is the mean difference of log units between stimuli) [37].

### Immunohistochemistry

At the end of the experiment (day 5), the L4/L5 segments of the spinal cord were exposed from the lumbar vertebral column via laminectomy and identified by tracing the dorsal roots from their respective dorsal root ganglia (DRG). Animals were perfused using 0.1 M phosphate buffered saline (PBS), followed by 4% paraformaldehyde (BBC Biochemical, WA, USA). Sampled tissues were post-fixed in 4% paraformaldehyde (BBC Biochemical) for 24 h at 4 °C, and then permeated with 30% sucrose (Sigma-Aldrich) in 0.1 M PBS for 48 h at 4 °C. Lumbar spinal cord segments were embedded in optimal cutting temperature (OCT) compound (Sakura Finetek, Tokyo, Japan) on dry ice. Using cryostat (Microm HM 505N; Thermo Scientific, MA, USA), frozen spinal cord segments were cut at a 20 μm thickness. Sections were collected in 0.1 M PBS at 4 °C. These sections were mounted on slide glass (Matsunami, Osaka, Japan) and incubated for 1 h in 0.2% Triton X-100 in 0.5% bovine serum albumin (BSA; BOVOGEN biologics, East Keilor, Australia) solution at room temperature (RT). After rinsing the slide glass with 0.5% BSA solution, double immunostaining using primary antibodies raised in different species was carried out. The sections were incubated overnight at 4 °C with primary antibodies: mouse anti-glial fibrillary acidic protein (GFAP 1:500; Millipore, CA, USA), rabbit anti-Iba-1 (1:500; Wako, Osaka, Japan). After rinsing in 0.5% BSA solution, sections were incubated at RT in dark for 1 h with secondary antibodies: anti-mouse and anti-rabbit-immunoglobulin G (IgG) labeled with Alexa Fluor 488 and Alexa Fluor 546 (1:200; Invitrogen, USA). Confocal laser microscope (LSM 5 Pascal, Zeiss, Oberkochen, Germany) was used to obtain immunofluorescent images. Quantitative analysis of GFAP and Iba-1 positive cells were performed on the spinal dorsal horn images taken through a 20X 0.5NA objective. ImageJ (https://imagej.nih.gov/ij/, National Institutes of Health, USA) was used for quantifying the GFAP positive cells and Iba-1 positive cells [22]. To quantify GFAP or Iba-1 positive cells, number of GFAP or Iba-1 positive cells from six lumbar spinal cord section images of each animal were averaged. Six animals were allocated in each group.

### ELISA

To investigate whether Buja administration decreases the quantity of TNF-α or IL-1β in the spinal cord, each cytokine was measured by enzyme linked immunosorbent assay (ELISA). The animals were sacrificed at the end of the experiment. After perfusion with 0.1 M PBS, the lumbar spinal cord segments were obtained as described above. Every collected tissue was stored in 1 ml RIPA buffer (Thermo Scientific) with protease inhibitor cocktail (Roche, Basel, Switzerland). Samples were assayed using a commercial rat TNF (BD OptEIA Set Rat TNF, BD biosciences, CA, USA) and mouse IL-1β (BD OptEIA Set mouse IL-1β, BD biosciences) ELISA kit following the manufacturer’s protocol. In brief, 1:10 dilution of serum was used for the quantification of both cytokines. Microtiter plates were coated overnight at 4 °C with anti-rat TNF or anti-mouse IL-1β monoclonal antibodies (mAbs). Each well was blocked with 10% fetal bovine serum (FBS; Gibco, Thermo Scientific) for 1 h at RT. Samples and standards were loaded after washing out FBS and incubated for 2 h at RT. Biotinylated anti-rat TNF and anti-mouse IL-1β mAbs were added for 1 h at RT. Streptavidin-horseradish peroxidase conjugate was treated and incubated for 30 min. TMB substrate solution (BD Bioscience) was treated for 30 mins, and then Stop solution was added. Washing each well with PBST (PBS with Tween-20; Sigma-Aldrich) was performed between every step. Optical density (O.D.) was measured at 450 nm with λ correction 570 nm. O.D. was measured in a microplate reader (Tecan). Total amount of protein in samples were measured using Bio-Rad protein assay kit (Bio-Rad, CA, USA). All results were normalized to the total amount of protein in each sample.

### Statistical analysis

All the data are presented as mean ± SEM (standard error of the mean). Statistical analysis and graphic works were performed with Prism 5.0 (GraphPad software, USA). One-way analysis of variance (ANOVA) or Two-way ANOVA followed by Bonferroni’s multiple comparison test was used for statistical analysis. In all cases, *p* < 0.05 was considered significant.

## Results

### Anti-allodynic effects of Buja in oxaliplatin-injected rats

**Fig. 1:**
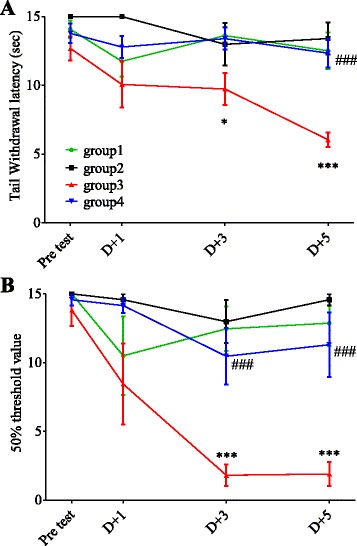
Inhibitory effect of Buja on cold and mechanical allodynia in oxaliplatin-injected rats. **a** Time course of tail withdrawal latency (TWL) in response to cold water (4 °C) stimuli. An oxaliplatin injection (group3: Oxaliplatin + DW) induced a significant decrease in TWL since day 3 (D + 3) compared to a vehicle injection (group1: Vehicle + DW). Daily oral administration of Buja (300 mg/kg) for 5 days following an oxaliplatin injection (group4: Oxaliplatin + Buja) significantly inhibited cold allodynia, whereas Buja had no effect on TWL in vehicle-injected rats (group2: Vehicle + Buja). **b** Time course of mechanical threshold. An oxaliplatin injection (group3) significantly decreased 50% threshold since day 3 compared to a vehicle injection (group1). Buja administration following an oxaliplatin injection (group4) significantly inhibited mechanical allodynia, whereas Buja had no effect on mechanical threshold in vehicle-injected rats (group2). *N* = 6 rats/group. Data are presented as mean ± SEM. * *p* < 0.05, *** *p* < 0.001, vs. group1; ^###^ *p* < 0.001, vs. group3, by two-way ANOVA followed by Bonferroni’s post-test

To investigate whether Buja alleviates oxaliplatin-induced neuropathic pain, cold and mechanical allodynia were assessed using tail immersion test and von Frey hair test, respectively, in randomly divided 4 groups of animals (see Methods). Significant cold allodynia sign (i.e. decreased TWL in response to cold stimuli) was observed since day 3 after an oxaliplatin (6 mg/kg, i.p.) injection (*p* < 0.05 at D + 3, *p* < 0.001 at D + 5, group1 vs. group3, Fig. 1a). Daily oral dministration of Buja (300 mg/kg) for 5 consecutive days following an oxaliplatin injection reversed such decrease in TWL to normal level (*p* < 0.001 at D + 5, group3 vs. group4, Fig. 1a). For mechanical allodynia, an oxaliplatin injection induced a significant decrease in 50% threshold since day 3 (*p* < 0.001 at D + 3 and D + 5, group1 vs. group3, Fig. 1b). Buja administration also reversed this mechanical allodynia sign to normal level (*p* < 0.001 at D + 3 and D + 5, group3 vs. group4, Fig. 1b). Buja administration in vehicle-injected rats (group2) showed no effect on behavioral responses to cold and mechanical stimuli (*p* > 0.05, vs group1, Fig. 1a and b). These results suggest that oral administration of Buja potently inhibits oxaliplatin-induced cold and mechanical allodynia in rats.

### Suppressive effect of Buja on activation of spinal astrocytes in oxaliplatin-injected rats

**Fig. 2:**
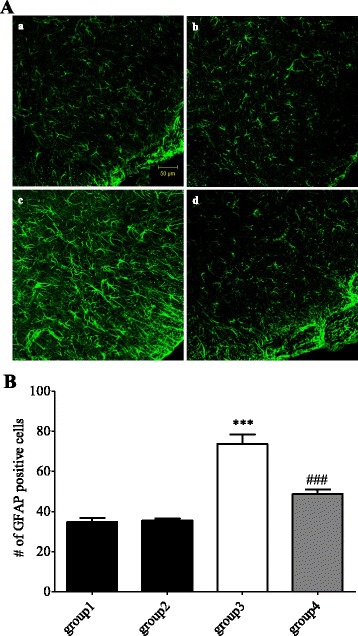
Buja attenuates the activation of spinal astrocytes in oxaliplatin-injected rats. **A** Representative images of GFAP positive cells in the spinal dorsal horn of group1: Vehicle + DW (*a*), group2: Vehicle + Buja (*b*), group3: Oxaliplatin + DW (*c*) and group4: Oxaliplatin + Buja (*d*). Note the increased number of GFAP positive cells and the altered morphology (somatic hypertrophy with thick processes) in the group3 (c), indicating activation of astrocytes. **B** Quantification result of GFAP positive cells. Six lumbar spinal cord section images from single animal were averaged. *N* = 6 rats/group. Data are presented as mean ± SEM. *** *p* < 0.001, vs. group1; ^###^ *p* < 0.001, vs. group3, by one-way ANOVA followed by Bonferroni’s post-test

To determine whether Buja suppresses glial activation in the dorsal horn of spinal cord after an oxaliplatin injection, activation of spinal glial cells (i.e. astrocytes and microglia) in laminae I-II of the dorsal horn was quantified using immunohistochemical analysis. As shown in Fig. 2, an oxaliplatin injection significantly increased GFAP-positive cells (astrocytes) in the spinal dorsal horn (*p* < 0.001, group1 vs. group3) and these cells in the group3 exhibited somatic hypertrophy with thick processes (Fig. 2a), which is also represented by enhanced intensity of immunoreactivity (Additional file 1: Figure S2), indicating oxaliplatin-induced activation of spinal astrocytes. Administration of Buja significantly reduced such activation of spinal astrocytes following an oxaliplatin injection (*p* < 0.001, group3 vs. group4, Fig. 2). Co-immunolabelings of GFAP (astrocytes) and Iba-1 (microglia) positive cells in the same spinal cord samples were performed and these cells were not co-localized (Additional file 2: Figure S3). The number of Iba-I positive cells (microglia) in the spinal dorsal horn is also increased following an oxaliplatin injection (*p* < 0.001, group1 vs. group3, Additional file 3: Figure S1) and these cells in the group3 showed amoeboid shapes with thick processes (Additional file 3: Figure S1A), which is represented by enhanced intensity of immunoreactivity (Additional file 1: Figure S2). However, Buja administration did not change such microglial activation, i.e. increased Iba-1 positive cells and altered morphology (*p* > 0.05, group3 vs. group4, Additional file 3: Figure S1). These results suggest that Buja suppresses activation of astrocytes in the spinal dorsal horn following an oxaliplatin injection without affecting microglial activation.

### Buja down-regulates the levels of spinal pro-inflammatory cytokines in oxaliplatin-injected rats

**Fig. 3:**
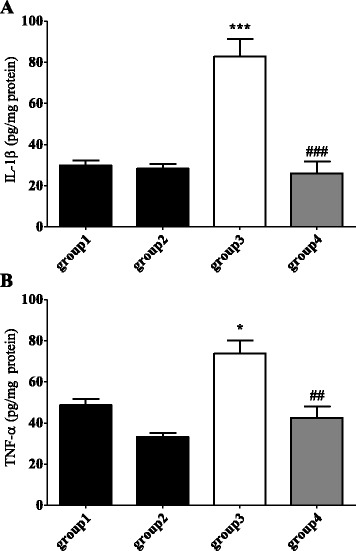
Buja suppresses the oxaliplatin-induced up-regulation of spinal pro-inflammatory cytokines. **a**, **b** Quantification of pro-inflammatory cytokines, IL-1β (**a**) and TNF-α (**b**), in the spinal cord. *N* = 6 rats/group. Data are presented as mean ± SEM. * *p* < 0.05, *** *p* < 0.001, vs. group1; ^##^ *p* < 0.01, ^###^ *p* < 0.001, vs. group3, by one-way ANOVA followed by Bonferroni’s post-test. group1: Vehicle + DW, group2: Vehicle + Buja, group3: Oxaliplatin + DW and group4: Oxaliplatin + Buja

Pro-inflammatory cytokines, IL-1β and TNF-α, were measured with ELISA carried out at the end of the experiment (day 5). As shown in Fig. 3, the levels of IL-1β and TNF-α in the spinal cord were significantly increased after an oxaliplatin injection (*p* < 0.001 for IL-1β, *p* < 0.05 for TNF-α, group1 vs. group3). Treatment of Buja reversed this up-regulation of IL-1β and TNF-α in the spinal cord to normal level (*p* < 0.001 for IL-1β, *p* < 0.01 for TNF-α, group3 vs. group4). These results suggest that Buja can suppress the oxaliplatin-induced increase in the levels of spinal pro-inflammatory cytokines.

## Discussion

Oxaliplatin, a widely used chemotherapeutic agent, is known to evoke peripheral neuropathy even after a single injection [1–3, 25]. Various kinds of experiments are still under way to divulge the exact mechanism of oxaliplatin-induced peripheral neuropathy and its optimal treatment method has not been developed yet [23, 38]. Although its exact pathophysiology is not clearly understood [23], accumulating evidences imply that activation of spinal glia, such as astrocytes and microglia, play an important role in oxaliplatin-induced neuropathic pain [21, 22, 25, 39]. Activated spinal glia were observed after oxaliplatin injection, and intrathecal injection of minocycline and fluorocitrate, which decrease the activation of astrocytes and microglia respectively, effectively attenuated neuropathic pain [22]. Activated glia are known to contribute to neuropathic pain by releasing pro-inflammatory cytokines such as IL-1β and TNF-α [13–15], and suppressing the activation of astrocytes and microglia down-regulated the expression of pro-inflammatory cytokines, which led to the alleviation of nerve injury-induced neuropathic pain [40, 41]. Intrathecal injection of pro-inflammatory cytokines such as IL-1β and TNF-α evoked hyperalgesia and allodynia in naive animals [42, 43]. Furthermore, blocking the action of IL-1β and TNF-α using IL-1 receptor antagonist and anti-TNF serum alleviated nerve injury-induced neuropathic pain [44, 45]. In an animal model of oxaliplatin-induced neuropathic pain, release of IL-1β and TNF-α from activated spinal glia were observed [39, 46]. Intrathecally injected A3 adenosine receptor agonists prevented the activation of astrocytes and the increase of pro-inflammatory cytokines in the spinal cord and significantly attenuated neuropathic pain [46]. Therefore, targeting spinal glial activation and pro-inflammatory cytokines could be an ideal strategy to attenuate oxaliplatin-induced neuropathic pain.

Buja is a generally used herbal medicine in East-Asia, such as Korea, Japan and China. It is also a key component in GBT, which has been traditionally used against cold-induced disorders based on the Sang Han Lun [25]. In addition, GBT showed anti-allodynic effect on oxaliplatin-induced neuropathic pain [25]. Recent experiments conducted on both human and animals have shown that Buja significantly improved cold or mechanical allodynia [26, 28, 30]. In the present study, five consecutive oral administrations of Buja (300 mg kg^−1^ per day) markedly alleviated oxaliplatin-induced cold and mechanical allodynia. These results strongly suggest that Buja has potent efficacy on oxaliplatin-induced neuropathic pain.

Our experiment showed that oral administration of Buja decreased the cold and mechanical allodynia via suppressing the activation of spinal astrocytes. This result is similar to that of Shibata et al. [26] where, Buja was suggested to control neuropathic pain via inhibition of activated astrocytes in nerve injury model. In neuropathic state, extracellular single-regulated kinase (ERK) pathways in astrocytes were activated by IL-1β and IL-18 from microglia and promoted the synthesis of pro-inflammatory cytokines, IL-1β and TNF-α [47, 48]. Buja directly affected inhibition of ERK 1/2-phosphorlation, which resulted in suppression of the spinal astrocytes [26]. However, in our previous study conducted with GBT (400 mg kg^−1^ for five days), GBT attenuated oxaliplatin-induced cold and mechanical allodynia by decreasing the activation of both spinal astrocytes and microglia [25]. Although both Buja and GBT alleviated oxaliplatin-induced cold and mechanical allodynia, Buja only attenuated the activation of spinal astrocytes, whereas GBT suppressed the activation of both astrocytes and microglia. Lorenzo et al. [22] mentioned that although the activation of both spinal astrocytes and microglia are important in oxaliplatin-induced neuropathic pain, down-regulating only astrocytes or microglia can lead to the attenuation of pain. In the action of suppressing the activated glia by GBT, activated astrocytes inhibition is achieved by Buja, and another component of GBT may be responsible for the attenuation of microglia. If any single medicinal herb, which has key effect, is omitted, a specific activity from complex formulas disappears [49]. Furthermore, studies of another component in GBT are on the progress.

Pro-inflammatory cytokines, such as IL-1β and TNF-α, released from activated glia, act on the spinal dorsal horn neurons and influence excitatory neurotransmissions [14, 15]. IL-1β, by inducing the phosphorylation of a specific N-methyl-D-aspartate (NMDA) receptor subunit, increases the influx of calcium ion through NMDA receptor channel and the production of nitric oxide. IL-1β also increases the generation of prostaglandin E2, which amplify the excitability of pain-projection neurons [14]. TNF-α, by enhancing the activation of α-Amino-3-hydroxy-5-methyl-4-isoxazolepropionic acid (AMPA) receptor, increases the excitatory post-synaptic currents frequency in the spinal dorsal horn [20]. These interactions between cytokines and neurons contribute to central sensitization [50] and further enhance neuropathic pain. In our result, the suppression of activated spinal astrocytes and the decrease of IL-1β and TNF-α levels in the spinal cord were coincided after treatment of Buja. GBT also down-regulated release of spinal pro-inflammatory cytokines [51]. Taken all together, our findings suggest that Buja strongly alleviate oxaliplatin-induced cold and mechanical allodynia via suppression of activated spinal astrocytes and down-regulation of pro-inflammatory cytokines.

## Conclusion

In conclusion, this study clearly demonstrated the relieving effect of Buja on oxaliplatin-induced cold and mechanical allodynia. Also, Buja significantly suppressed the activated spinal astrocytes and down-regulated pro-inflammatory cytokines after an oxaliplatin injection. These results altogether suggest that Buja may be an effective alternative to treat oxaliplatin-induced neuropathic pain.

## Additional files

Additional file 1: Figure S2. Intensity of immunoreactivity (IM) of GFAP and Iba-1 positive cells. Intensity of IM of GFAP and Iba-1 positive cells increased in group3. In group4, IM of GFAP positive cells were significantly decreased, whereas little changes of Iba-1 positive cells IM was observed. Data indicate that relative mean immunofluorescence intensity of a single cell (*n* = 10). Data are presented as mean ± SEM. *** *p* < 0.001, vs. group1; ^###^ *p* < 0.001, vs. group3, by one-way ANOVA followed by Bonferroni’s post-test. group1: Vehicle + DW, group2: Vehicle + Buja, group3: Oxaliplatin + DW and group4: Oxaliplatin + Buja. (PDF 7 kb)
Additional file 2: Figure S3. Representative co-immunolabeling image of GFAP and Iba-1 positive cells in the spinal dorsal horn. Co-immunolabeling in the same section showed the spatially different distribution of astrocytes (GFAP-positive cells) and microglia (Iba-1 positive cells) in the spinal dorsal horn (a). Separated images for astrocytes (b) and microglia (c) were also presented, respectively. (PDF 236 kb)
Additional file 3: Figure S1. Spinal microglia activation was not suppressed by Buja. (A) Representative images of Iba-1 positive cells in the spinal dorsal horn of group1: Vehicle + DW (a), group2: Vehicle + Buja (b), group3: Oxaliplatin + DW (c) and group4: Oxaliplatin + Buja (d). Note the increased number of Iba-1 positive cells and the altered morphology (somatic hypertrophy with thick processes) in the group3 (c), indicating activation of microglia. (B) Quantification result of Iba-1 positive cells. Six lumbar spinal cord section images from single animal were averaged. *N* = 6 rats/group. Data are presented as mean ± SEM. *** *p* < 0.001, vs. group1; ^###^ *p* < 0.001, vs. group3, by one-way ANOVA followed by Bonferroni’s post-test. (PDF 260 kb)

## Abbreviations

AMPA: α-amino-3-hydroxy-5-methyl-4-isoxazolepropionic acid
ANOVA: Analysis of variance
BSA: Bovine serum albumin
CIPN: Chemotherapy-induced neuropathic pain
DRG: Dorsal root ganglia
DW: Distilled water
ELISA: Enzyme-linked immunosorbent assay
ERK: Extracellular single-regulated kinase
FBS: Fetal bovine serum
GBT: Gyejigachulbu-Tang
GFAP: Glial fibrillary acidic protein
IgG: Immunoglobulin G
IL-1β: Interleukin-1β
IM: Immunoreactivity
mAbs: Monoclonal antibodies
NMDA: N-methyl-D-aspartate
O.D.: Optical density
OCT: Optimal cutting temperature
PBS: Phosphate buffered saline
PBST: Phosphate buffered saline with Tween 20
RT: Room temperature
SD rat: Sprauge-Dawley rat
SEM: Standard error and mean
TNF-α: Tumor necrosis factor-α
TWL: Tail withdrawal latency

## References

[1] Windebank AJ, Grisold W (2008). Chemotherapy-induced neuropathy. J Peripher Nerv Syst.

[2] McWhinney SR, Goldberg RM, McLeod HL (2009). Platinum Neurotoxicity Pharmacogenetics. Mol Cancer Ther.

[3] Raymond E, Faivre S, Woynarowski JM, Chaney SG (1998). Oxaliplatin: mechanism of action and antineoplastic activity. Semin Oncol.

[4] Formiga MN, Fanelli MF, Dettino ALA, Nicolau UR, Cavicchioli M, Lima ENP, de Mello CAL (2016). Is early response by 18 F-2-fluoro-2-deoxy-D-glucose positron emission tomography-computed tomography a predictor of long-term outcome in patients with metastatic colorectal cancer?. J Gastrointest Oncol.

[5] Yoshino T, Uetake H, Tsuchihara K, Shitara K, Yamazaki K, Oki E, Sato T, Naitoh T, Komatsu Y, Kato T (2016). PARADIGM study: A multicenter, randomized, phase III study of 5-fluorouracil, leucovorin, and oxaliplatin (mFOLFOX6) plus panitumumab or bevacizumab as first-line treatment in patients with RAS (KRAS/NRAS) wild-type metastatic colorectal cancer. ASCO Annu Meet Proc.

[6] Extra JM, Espie M, Calvo F, Ferme C, Mignot L, Marty M (1990). Phase I study of oxaliplatin in patients with advanced cancer. Cancer Chemother Pharmacol.

[7] Lehky TJ, Leonard GD, Wilson RH, Grem JL, Floeter MK (2004). Oxaliplatin-induced neurotoxicity: Acute hyperexcitability and chronic neuropathy. Muscle Nerve.

[8] Pasetto LM, D’Andrea MR, Rossi E, Monfardini S (2006). Oxaliplatin-related neurotoxicity: How and why?. Crit Rev Oncol Hematol.

[9] Wolf S, Barton D, Kottschade L, Grothey A, Loprinzi C (2008). Chemotherapy-induced peripheral neuropathy: prevention and treatment strategies. Eur J Cancer.

[10] Gamelin L, Boisdron-Celle M, Delva R, Guérin-Meyer V, Ifrah N, Morel A, Gamelin E (2004). Prevention of Oxaliplatin-Related Neurotoxicity by Calcium and Magnesium Infusions A Retrospective Study of 161 Patients Receiving Oxaliplatin Combined with 5-Fluorouracil and Leucovorin for Advanced Colorectal Cancer. Clin Cancer Res.

[11] Mariani G, Garrone O, Granetto C, Numico G, LaCiura P, Grecchi G, DiCostanzo G, Merlano M (2000). Oxaliplatin induced neuropathy: could gabapentin be the answer. Proc Am Soc Clin Oncol.

[12] Serpell M, Group NPS (2002). Gabapentin in neuropathic pain syndromes: a randomised, double-blind, placebo-controlled trial. Pain.

[13] Scholz J, Woolf CJ (2007). The neuropathic pain triad: neurons, immune cells and glia. Nat Neurosci.

[14] Milligan ED, Watkins LR (2009). Pathological and protective roles of glia in chronic pain. Nat Rev Neurosci.

[15] Benarroch EE (2010). Central neuron-glia interactions and neuropathic pain Overview of recent concepts and clinical implications. Neurology.

[16] Lim B-S, Moon HJ, Li DX, Gil M, Min JK, Lee G, Bae H, Kim SK, Min B-I (2013). Effect of bee venom acupuncture on oxaliplatin-induced cold allodynia in rats. Evid Based Complement Alternat Med.

[17] Mika J, Osikowicz M, Rojewska E, Korostynski M, Wawrzczak-Bargiela A, Przewlocki R, Przewlocka B (2009). Differential activation of spinal microglial and astroglial cells in a mouse model of peripheral neuropathic pain. Eur J Pharmacol.

[18] Vallejo R, Tilley DM, Vogel L, Benyamin R (2010). The role of glia and the immune system in the development and maintenance of neuropathic pain. Pain Pract.

[19] DeLeo JA, Colburn RW: Proinflammatory cytokines and glial cells: Their role in neuropathic pain. In: Cytokines and Pain. edn. Edited by Watkins LR, Maier SF. Basel: Birkhäuser Basel; 1999;159–181.

[20] Kawasaki Y, Zhang L, Cheng J-K, Ji R-R (2008). Cytokine mechanisms of central sensitization: distinct and overlapping role of interleukin-1β, interleukin-6, and tumor necrosis factor-α in regulating synaptic and neuronal activity in the superficial spinal cord. J Neurosci.

[21] Di Cesare Mannelli L, Pacini A, Bonaccini L, Zanardelli M, Mello T, Ghelardini C (2013). Morphologic Features and Glial Activation in Rat Oxaliplatin-Dependent Neuropathic Pain. J Pain.

[22] Di Cesare Mannelli L, Pacini A, Micheli L, Tani A, Zanardelli M, Ghelardini C (2014). Glial role in oxaliplatin-induced neuropathic pain. Exp Neurol.

[23] Carozzi V, Canta A, Chiorazzi A (2015). Chemotherapy-induced peripheral neuropathy: What do we know about mechanisms?. Neurosci Lett.

[24] Vichaya EG, Chiu GS, Krukowski K, Lacourt TE, Kavelaars A, Dantzer R, Heijnen CJ, Walker AK (2015). Mechanisms of chemotherapy-induced behavioral toxicities. Front Neurosci.

[25] Ahn B-S, Kim S-K, Kim HN, Lee J-H, Lee J-H, Hwang DS, Bae H, Min B-I, Kim SK (2014). Gyejigachulbu-Tang Relieves Oxaliplatin-Induced Neuropathic Cold and Mechanical Hypersensitivity in Rats via the Suppression of Spinal Glial Activation. Evid Based Complement Alternat Med.

[26] Shibata K, Sugawara T, Fujishita K, Shinozaki Y, Matsukawa T, Suzuki T, Koizumi S (2011). The Astrocyte-Targeted Therapy by Bushi for the Neuropathic Pain in Mice. PLoS ONE.

[27] Schröder S, Beckmann K, Franconi G, Meyer-Hamme G, Friedemann T, Greten HJ, Rostock M, Efferth T (2013). Can medical herbs stimulate regeneration or neuroprotection and treat neuropathic pain in chemotherapy-induced peripheral neuropathy?. Evid Based Complement Alternat Med.

[28] Suzuki Y, Goto K, Ishige A, Komatsu Y, Kamei J (1999). Antinociceptive Effect of < I > Gosha-jinki-gan</I > a < I > Kampo</I > Medicine, in Streptozotocin-Induced Diabetic Mice. Jpn J Pharmacol.

[29] Nakanishi M, Arimitsu J, Kageyama M, Otsuka S, Inoue T, Nishida S, Yoshikawa H, Kishida Y (2012). Efficacy of Traditional Japanese Herbal Medicines—Keishikajutsubuto (TJ-18) and Bushi-matsu (TJ-3022)—Against Postherpetic Neuralgia Aggravated by Self-Reported Cold Stimulation: A Case Series. J Altern Complement Med.

[30] Yamada T, Kan H, Matsumoto S, Koizumi M, Sasaki J, Tani A, Yokoi K, Uchida E (2012). [Reduction in oxaliplatin-related neurotoxicity by the administration of Keishikajutsubuto (TJ-18) and powdered processed aconite root]. Gan To Kagaku Ryoho.

[31] Zimmermann M (1983). Ethical guidelines for investigations of experimental pain in conscious animals. Pain.

[32] Li D, Lee Y, Kim W, Lee K, Bae H, Kim KS (2015). Analgesic Effects of Bee Venom Derived Phospholipase A2 in a Mouse Model of Oxaliplatin-Induced Neuropathic Pain. Toxins.

[33] Ling B, Coudoré-Civiale M-A, Balayssac D, Eschalier A, Coudoré F, Authier N (2007). Behavioral and immunohistological assessment of painful neuropathy induced by a single oxaliplatin injection in the rat. Toxicology.

[34] Na HS, Han JS, Ko KH, Hong SK (1994). A behavioral model for peripheral neuropathy produced in rat’s tail by inferior caudal trunk injury. Neurosci Lett.

[35] Kim SK, Park JH, Bae SJ, Kim JH, Hwang BG, Min B-I, Park DS, Na HS (2005). Effects of electroacupuncture on cold allodynia in a rat model of neuropathic pain: Mediation by spinal adrenergic and serotonergic receptors. Exp Neurol.

[36] Chaplan SR, Bach FW, Pogrel JW, Chung JM, Yaksh TL: Quantitative assessment of tactile allodynia in the rat paw. J Neurosci Methods. 1994;53(1):55–63. 10.1016/0165-0270(94)90144-9 7990513

[37] Dixon WJ (1980). Efficient Analysis of Experimental Observations. Annu Rev Pharmacol Toxicol.

[38] Cavaletti G, Marmiroli P (2015). Chemotherapy-induced peripheral neurotoxicity. Curr Opin Neurol.

[39] Yoon S-Y, Robinson CR, Zhang H, Dougherty PM (2013). Spinal astrocyte gap junctions contribute to oxaliplatin-induced mechanical hypersensitivity. J Pain.

[40] Hu C, Zhang G, Zhao Y-T (2014). Fucoidan attenuates the existing allodynia and hyperalgesia in a rat model of neuropathic pain. Neurosci Lett.

[41] Chu L-W, Chen J-Y, Wu P-C, Wu B-N (2015). Atorvastatin Prevents Neuroinflammation in Chronic Constriction Injury Rats through Nuclear NFκB Downregulation in the Dorsal Root Ganglion and Spinal Cord. ACS Chem Neurosci.

[42] Kwon M-S, Shim E-J, Seo Y-J, Choi S-S, Lee J-Y, Lee H-K, Suh H-W (2005). Differential Modulatory Effects of Cholera Toxin and Pertussis Toxin on Pain Behavior Induced by TNF-a, lnterleukin-1β and Interferon- Injected Intrathecally. Arch Pharm Res.

[43] D-h Y, Wang H, Jeong S-J (2008). Exogenous tumor necrosis factor-α rapidly alters synaptic and sensory transmission in the adult rat spinal cord dorsal horn. J Neurosci Res.

[44] Sweitzer S, Martin D, DeLeo JA (2001). Intrathecal interleukin-1 receptor antagonist in combination with soluble tumor necrosis factor receptor exhibits an anti-allodynic action in a rat model of neuropathic pain. Neuroscience.

[45] Winkelstein BA, Rutkowski MD, Sweitzer SM, Pahl JL, DeLeo JA (2001). Nerve injury proximal or distal to the DRG induces similar spinal glial activation and selective cytokine expression but differential behavioral responses to pharmacologic treatment. J Comp Neurol.

[46] Janes K, Wahlman C, Little JW, Doyle T, Tosh DK, Jacobson KA, Salvemini D (2015). Spinal neuroimmune activation is independent of T-cell infiltration and attenuated by A 3 adenosine receptor agonists in a model of oxaliplatin-induced peripheral neuropathy. Brain Behav Immun.

[47] Ji R-R, Gereau Iv RW, Malcangio M, Strichartz GR (2009). MAP kinase and pain. Brain Res Rev.

[48] Zhuang Z-Y, Gerner P, Woolf CJ, Ji R-R (2005). ERK is sequentially activated in neurons, microglia, and astrocytes by spinal nerve ligation and contributes to mechanical allodynia in this neuropathic pain model. Pain.

[49] Hosoya E (1988). Scientific reevaluation of Kampo prescriptions using modern technology.

[50] Ren K, Dubner R (2008). Neuron-glia crosstalk gets serious: Role in pain hypersensitivity. Curr Opin Anaesthesiol.

[51] Kim HN (2015). Gyejigachulbu-tang suppresses oxaliplatin-induced neuropathic mechanical allodynia in rats via modulating spinal TNF- α.

