# Supplementary figures and images for: Anti-allodynic effect of Buja in a rat model of oxaliplatin-induced peripheral neuropathy via spinal astrocytes and pro-inflammatory cytokines suppression
Source: BMC Complement Altern Med. 2017 Jan 14;17:48. doi: 10.1186/s12906-017-1556-z (PMC5237549; doi:10.1186/s12906-017-1556-z)

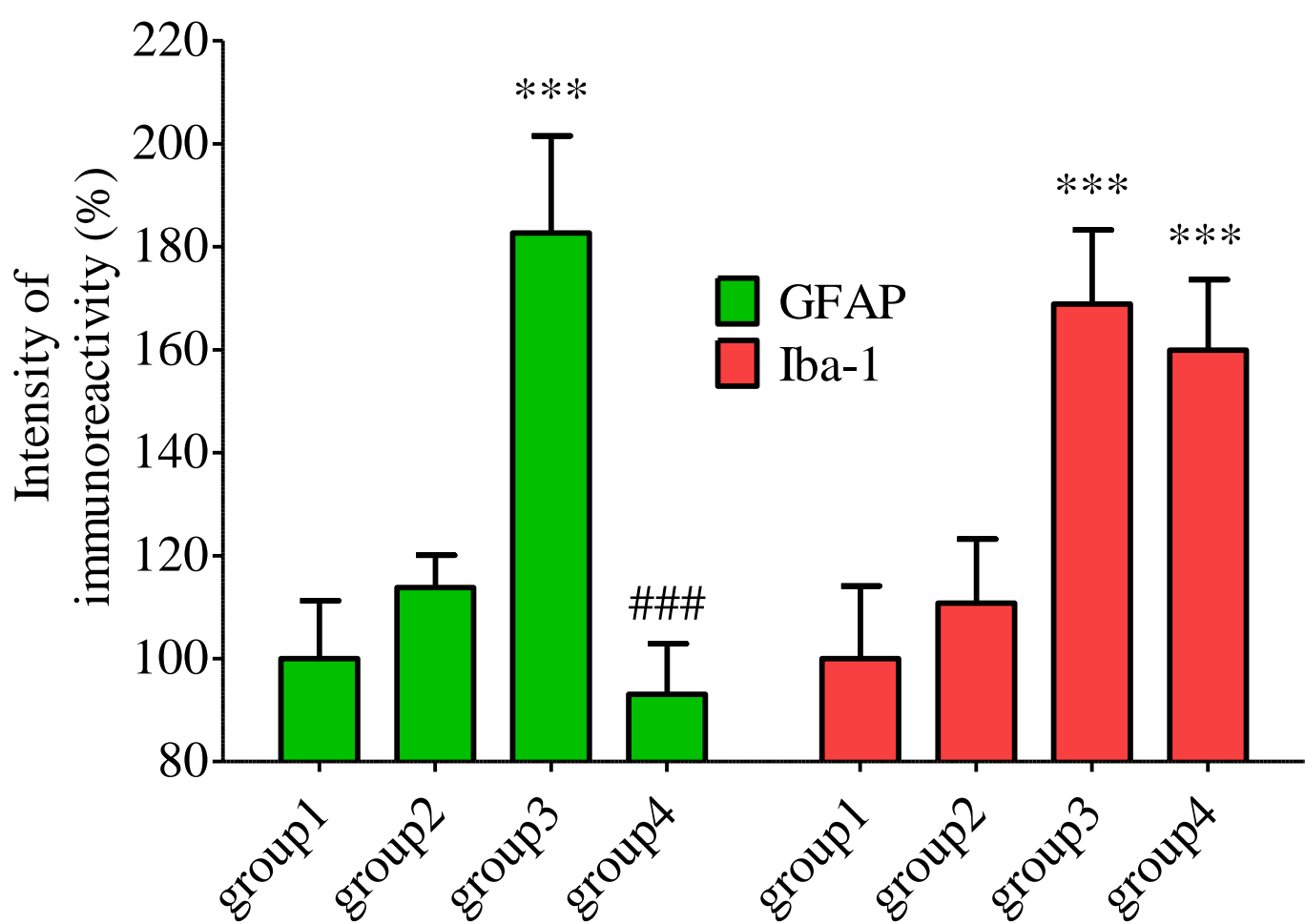

Supplement: Additional file 1: Figure S2. — Intensity of immunoreactivity (IM) of GFAP and Iba-1 positive cells. Intensity of IM of GFAP and Iba-1 positive cells increased in group3. In group4, IM of GFAP positive cells were significantly decreased, whereas little changes of Iba-1 positive cells IM was observed. Data indicate that relative mean immunofluorescence intensity of a single cell (n = 10). Data are presented as mean ± SEM. *** p < 0.001, vs. group1; ### p < 0.001, vs. group3, by one-way ANOVA followed by Bonferroni’s post-test. group1: Vehicle + DW, group2: Vehicle + Buja, group3: Oxaliplatin + DW and group4: Oxaliplatin + Buja. (PDF 7 kb) [file 12906_2017_1556_MOESM1_ESM.pdf]

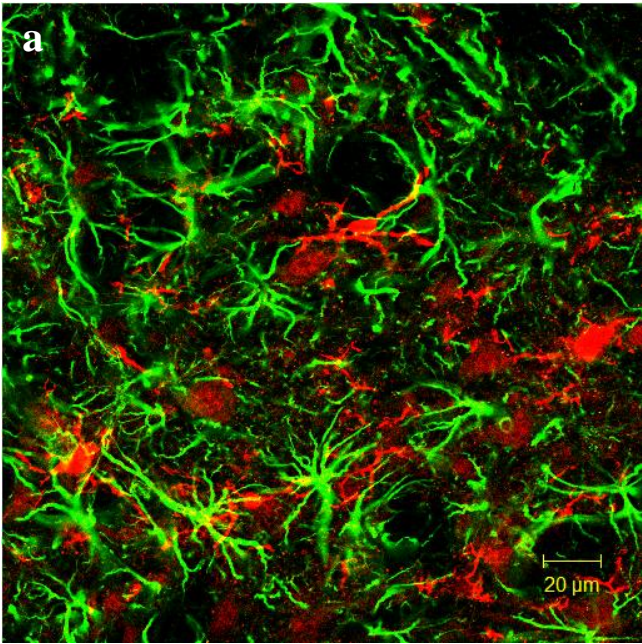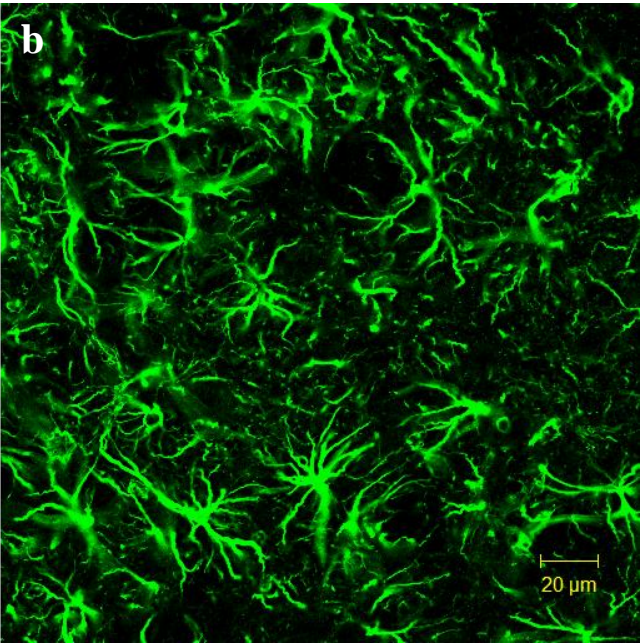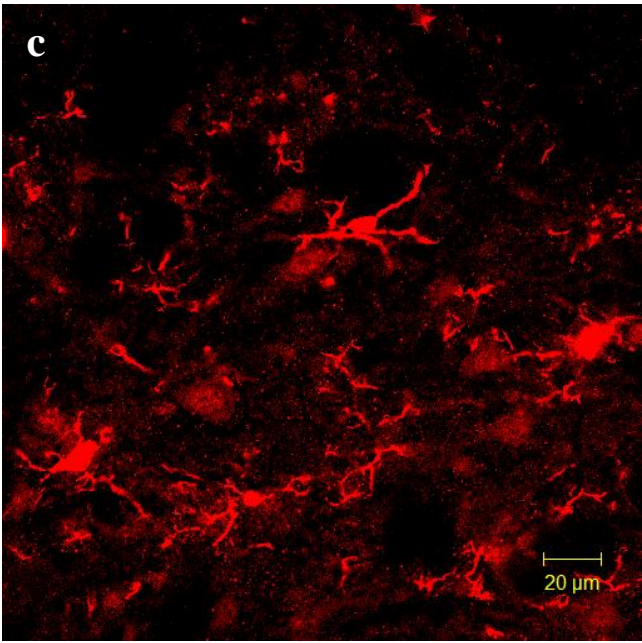

Supplement: Additional file 2: Figure S3. — Representative co-immunolabeling image of GFAP and Iba-1 positive cells in the spinal dorsal horn. Co-immunolabeling in the same section showed the spatially different distribution of astrocytes (GFAP-positive cells) and microglia (Iba-1 positive cells) in the spinal dorsal horn (a). Separated images for astrocytes (b) and microglia (c) were also presented, respectively. (PDF 236 kb) [file 12906_2017_1556_MOESM2_ESM.pdf]

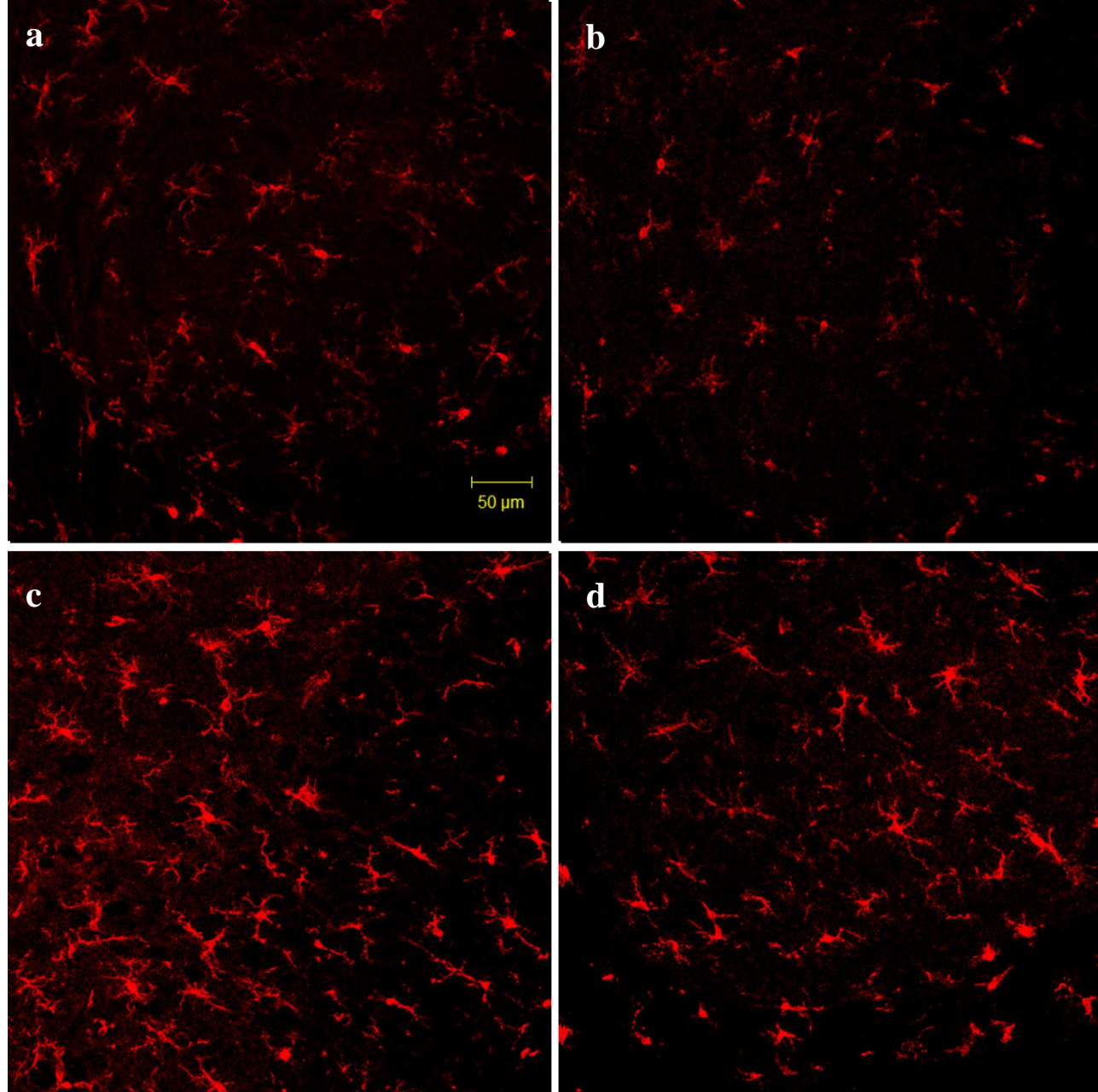

**A**

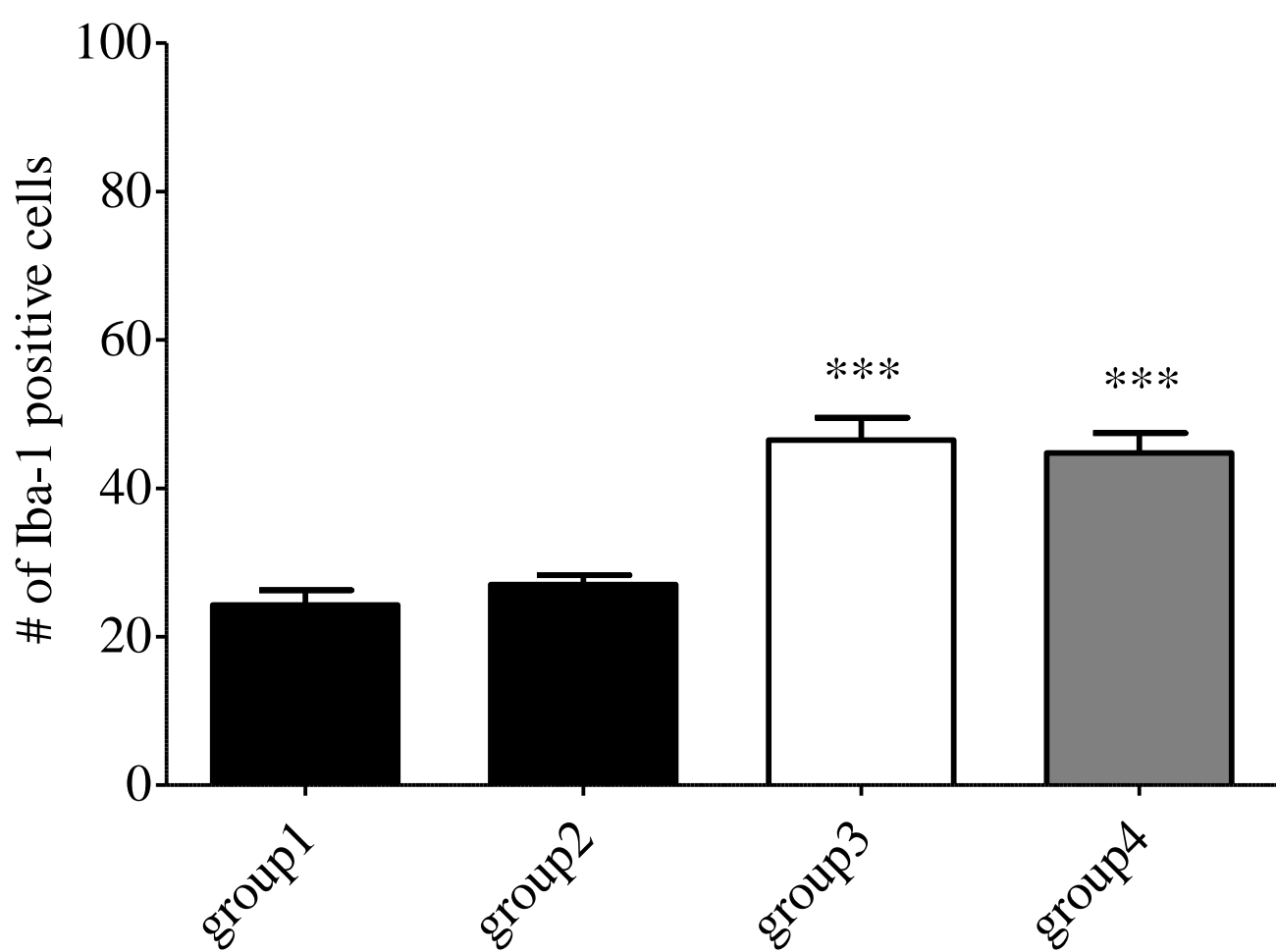

**B**

Supplement: Additional file 3: Figure S1. — Spinal microglia activation was not suppressed by Buja. (A) Representative images of Iba-1 positive cells in the spinal dorsal horn of group1: Vehicle + DW (a), group2: Vehicle + Buja (b), group3: Oxaliplatin + DW (c) and group4: Oxaliplatin + Buja (d). Note the increased number of Iba-1 positive cells and the altered morphology (somatic hypertrophy with thick processes) in the group3 (c), indicating activation of microglia. (B) Quantification result of Iba-1 positive cells. Six lumbar spinal cord section images from single animal were averaged. N = 6 rats/group. Data are presented as mean ± SEM. *** p < 0.001, vs. group1; ### p < 0.001, vs. group3, by one-way ANOVA followed by Bonferroni’s post-test. (PDF 260 kb) [file 12906_2017_1556_MOESM3_ESM.pdf]
